# Insulin-Like Growth Factor Binding Protein-6 Alters Skeletal Muscle Differentiation of Human Mesenchymal Stem Cells

**DOI:** 10.1155/2017/2348485

**Published:** 2017-09-14

**Authors:** Doaa Aboalola, Victor K. M. Han

**Affiliations:** ^1^Departments of Anatomy and Cell Biology, Western Ontario University, London, ON, Canada; ^2^Children's Health Research Institute, Western Ontario University, London, ON, Canada; ^3^Lawson Health Research Institute, Western Ontario University, London, ON, Canada; ^4^King Abdullah International Medical Research Center, National Guard Health Affairs, Jeddah, Saudi Arabia; ^5^Departments of Paediatrics, Schulich School of Medicine & Dentistry, Western Ontario University, London, ON, Canada

## Abstract

Insulin-like growth factor binding protein-6 (IGFBP-6), the main regulator of insulin-like growth factor-2 (IGF-2), is a component of the stem cell niche in developing muscle cells. However, its role in muscle development has not been clearly defined. In this study, we investigated the role of IGFBP-6 in muscle commitment and differentiation of human mesenchymal stem cells derived from the placenta. We showed that placental mesenchymal stem cells (PMSCs) have the ability to differentiate into muscle cells when exposed to a specific culture medium by expressing muscle markers Pax3/7, MyoD, myogenin, and myosin heavy chain in a stage-dependent manner with the ultimate formation of multinucleated fibers and losing pluripotency-associated markers, OCT4 and SOX2. The addition of IGFBP-6 significantly increased pluripotency-associated markers as well as muscle differentiation markers at earlier time points, but the latter decreased with time. On the other hand, silencing IGFBP-6 decreased both pluripotent and differentiation markers at early time points. The levels of these markers increased as IGFBP-6 levels were restored. These findings indicate that IGFBP-6 influences MSC pluripotency and myogenic differentiation, with more prominent effects observed at the beginning of the differentiation process before muscle commitment.

## 1. Introduction

Unlike embryonic stem cells which are derived from the early embryo, placental mesenchymal stem cells (PMSCs) are derived from human placentae that are usually discarded following delivery, and therefore a readily available and noncontroversial source of adult stem cells for possible use in tissue regenerative therapies in human patients [1–3]. Placental mesenchymal stem cells are available in large numbers and capable of differentiating into cells of all three germ layers depending on the type and concentration of niche factors to which the cells are exposed to *in vitro*. The pathways activated by these cells during differentiation into specific mesodermal cell types illustrate the mechanisms by which these cells differentiate *in vitro* and *in vivo* and may provide important information on the developmental processes of tissues and organs during embryogenesis and in the adult.

Skeletal muscle development is a highly coordinated stepwise process utilizing a series of transcriptional factors, and structural and enzymatic proteins expressed to mark the different stages of skeletal muscle development. During myogenesis, committed progenitors differentiate into muscle lineage by upregulating the myogenic regulatory factors (MRFs) as well as muscle commitment transcription factors (Pax3 and Pax7), followed by the expression of early muscle cell markers (MyoD and myogenin) [4]. After commitment, the cells start to fuse together to form multinucleated fibers and express muscle-specific proteins, such as myosin heavy chain (MHC) [4]. It is believed that in recovery and regeneration after muscle injury in the adult, this process is recapitulated.

Mesenchymal stem cells isolated from bone marrow have the ability to differentiate into myocytes [5, 6]. However, these cells have limited availability and do not have the ability to form fused skeletal muscle *in vitro* [7, 8]. Adipose-derived stem cells are another source of stem cells that can differentiate into skeletal muscle [9]. Although these cells are readily available, they have limited muscle recovery [10]. Therefore, the need to find a stem cell population that will eliminate the problems related to other stem cells was our main priority.

The insulin-like growth factor (IGF) family of peptides regulates cell growth, differentiation, and the maintenance of cell survival through several signal transduction pathways [11]. This family includes two IGF peptides, IGF-1 and IGF-2, three cell surface receptors, type-1 and type-2 IGF receptors, insulin and hybrid receptors, and six IGF binding proteins (IGFBPs) [4]. IGF-1 and IGF-2 are circulating and intercellular peptides that function as potent mitogens for many different cell types, which are mediated by binding to IGF-1R, a membrane receptor tyrosine kinase [12]. IGFBPs are carriers for IGFs in the circulation and in the extracellular fluid compartment [13], protecting them from degradation [12, 14], delivering them to specific tissues, and modulating the biological actions of IGFs. IGFBP-6 is a 30 kDa secreted protein, and unlike other IGFBPs, has a significantly higher affinity (~70–100-fold) for IGF-2 than IGF-1 [15, 16]. IGFBP-6 has been demonstrated to modulate IGF-2 activity via inhibiting IGF-2 binding to the IGF-1R or directly independent of IGF-2 binding to the receptor [17, 18]. IGF binding proteins, including IGFBP-6, are secreted into the extracellular environment where they interact with IGFs. They are also localized intracellularly suggesting that IGFBPs may have biological actions independent of IGFs [19].

The IGF family has been shown previously to play a major role in muscle development. IGF-1R knockout mice die soon after birth due to breathing difficulties due to lack of functional respiratory muscles [20, 21]. IGF-2 is expressed abundantly in the developing skeletal muscle and is a major factor for muscle growth, differentiation, and regeneration [22]. When IGF-2 is knocked down, myogenesis does not occur [22]. During development, IGFBP-6 is expressed abundantly in developing muscle cells and is also required for myogenesis [22]. Previous studies in our laboratory have described IGF-independent functions of IGFBP-6 by interacting with Ku proteins in regulating cell fate in a skeletal muscle cell line [23]. Also, another study showed that IGFBP-6 inhibits angiogenesis but promotes migration in an IGF-independent manner [17].

To our knowledge, the biological roles of IGFBP-6 in the differentiation of stem cells into the muscle lineage have not been reported. In this study, we determined if PMSCs can differentiate into skeletal muscle when exposed to muscle differentiation promoting conditions and then characterized the effects of IGFBP-6 on the differentiation of PMSCs into skeletal muscle.

## 2. Materials and Methods

### 2.1. Isolation of PMSCs

PMSC isolation and experiments were conducted in accordance with the approval from the Health Sciences Research Ethics Board of Western University. Informed consent was obtained from healthy women undergoing therapeutic termination of pregnancy, and the PMSCs used in this study were isolated from 15 weeks preterm placental tissues. After surgery, chorionic villi were dissected, washed, minced with surgical scissors and forceps, and then subjected to enzymatic digestion with collagenase IV (369 IU/mg), hyaluronidase (999 IU/mg) (Sigma-Aldrich, Oakville, ON), and DNase I (2000 IU/mg) (Hoffmann-LaRoche, Mississauga, ON) for 10 minutes at room temperature, followed by 0.05% trypsin (Gibco/Invitrogen, Mississauga, ON) for 5 minutes at room temperature. The sample was then washed for 10 minutes with 10% FBS in DMEM/F12 medium, and the resulting single cell suspension was separated by density centrifugation over a Percoll (Sigma-Aldrich, Oakville, ON) discontinuous gradient using a modified protocol by Worton et al. [24].

### 2.2. Cell Culture

Cells from Percoll gradient fractions number 3 and number 4 were plated on to T75 flasks, cultured, and maintained using DMEM/F12 media supplemented with 15% FBS serum (Gibco/Invitrogen, Mississauga, ON) and FGF-2 (50 ng/mL) (Sigma-Aldrich, Oakville, ON) containing 100 U/mL penicillin, 100 *μ*g/mL streptomycin, and 0.25 *μ*g/mL amphotericin-B. The nonadherent cells were discarded at the time of media change, which was performed every 72 hours. The adherent cells were cultured until they reach 90% confluence. Cells were then passaged 1 : 2 approximately once per week using 0.05% trypsin for 10 min at 37°C for 3 passages. Fourth passaged cells were stored at −80°C in 1 mL of freezing media (30% FBS and 10% DMSO in DMEM/F12 media). When needed, vials were thawed and cells were resuspended in normal culture media (25 ng/mL FGF-2 and 15% FBS in DMEM/F12). Only PMSCs of passage 3 or 4 were used in the experiments.

### 2.3. Flow Cytometry Analysis

Cells were trypsinized for 10 minutes using recombinant trypsin (TrypLE EXpress, Gibco/Invitrogen, Mississauga, ON) diluted 1 : 1 in PBS, at 37°C. After the cells were detached from the flask, trypsin was neutralized with 10% FBS in DMEMF/12 medium, cells were washed and incubated for one hour with fluorochrome-labeled primary antibody against MSC markers. CD73 (number 550256) (BD Pharmingen, San Jose, CA), PE-conjugated CD105 (number 12-1057-73) (eBioscience, San Diego, CA), and CD-117/c-Kit (sc-13508) (Santa Cruz Biotechnology, Dallas, TX) were used (Supplementary Figure 1 available online at https://doi.org/10.1155/2017/2348485).

### 2.4. Muscle Differentiation

Cells were plated in the presence of the muscle growth media (fetal bovine serum 0.05 mL/mL, fetuin 50 *μ*g/mL, epidermal growth factor 10 ng/mL, basic fibroblast growth factor 1 ng/mL, insulin 10 *μ*g/mL, and dexamethasone 0.4 *μ*g/mL) for 48 hours before changing to the skeletal muscle differentiation media, which is serum-free medium containing 10 *μ*g/mL insulin (PromoCell, Heidelberg, Germany). Cells were grown in six-well plates in a standard tissue culture incubator at 37°C in 5% CO_2_.

### 2.5. IGFBP-6

Recombinant Human IGFBP-6 (ProSpec, East Brunswick, NJ) was resuspended in sterile MilliQ-H_2_O and added to the media at a concentration of 375 ng/mL. IGFBP-6 was added every 3 days at the time of media change. IGFBP-6 concentration was determined by a dose-response experiment using PMSCs in muscle differentiation media (Supplementary Figure 2A). IGFBP-6 was added every 3 days at the time of media change because that was the time it took for IGFBP-6 secreted levels to be lower than the control (Supplementary Figure 2B).

### 2.6. Downregulation of IGFBP-6 Expression by siRNA

To silence the endogenous IGFBP-6 expression, IGFBP6 siRNA (h) with a pool of 3 target-specific 19–25 nt siRNAs was used (Santa Cruz Biotechnology, Dallas, TX). 8 *μ*L of Lipofectamine (Invitrogen, Mississauga, ON) with either 8 *μ*L of scrambled or IGFBP-6 siRNA was added to 100 *μ*L of DMEM/F12 media (Invitrogen, Mississauga, ON) for 40 minutes at room temperature; the concentration of siRNA was 80 nM. The siRNA solution was then added to the 60% confluent cells and incubated for 5 hours at 37°C. Muscle growth media (1.5 mL) was added to the cells for 48 hours, and then it was replaced with 2 mL of muscle differentiation media. New siRNA was added every 3 days during the change of media, and the experiment was performed for 7 days.

### 2.7. Immunocytochemistry

PMSCs were grown and differentiated on glass cover slips, stained with primary antibodies, and incubated at 4°C overnight. The primary antibodies were washed using 0.1% Tween-20 in PBS (3 times for 5 minutes), and cells were then incubated in the dark with the secondary antibody. The secondary antibody was washed 0.1% Tween-20 in PBS, and the nuclear stain was added for 7 minutes and then rinsed. The cover slips were mounted for 2 hours, and images were taken using a Zeiss confocal microscope. Each antibody was performed in triplicate.

### 2.8. Immunoblotting

Following experiment completion, each cell lysate containing 20 *μ*g of protein was added to 6x SDS gel loading buffer (1% *β*-mercaptoethanol, 1% SDS, 30% glycerol, 0.0012% bromophenol blue, Tris-HCl 0.28 M, and pH 6.8). Samples were boiled for 5 minutes at 95°C, then placed on ice for 3 minutes, and centrifuged at 3000 rpm for 20 seconds before loading. Samples were resolved by molecular weight using 10% SDS polyacrylamide gels and then transferred onto polyvinyldenefluoride (PVDF) membranes (Bio-Rad, Hercules, California) using a Trans-Blot Turbo (Bio-Rad, Hercules, California) with an optimized protocol depending on the protein size. Membranes were blocked with 5% nonfat dry milk, gently shaked for 1 hour at room temperature in Tris-HCl buffer saline pH 8.0 with 0.1% Tween-20 (TBS-T). Blots were then washed with TBS-T (3x for 10 min) followed by incubation at 4°C overnight with specific primary antibodies in 5% BSA or 5% nonfat dry milk in TBS-T following the manufacturer's protocol. Then membranes were washed and incubated for 1 hour at room temperature with the corresponding secondary HRP antibody. Resolved protein bands were detected using chemiluminescence, and images were taken using the VersaDoc Imager (Bio-Rad, Hercules, California). Western blots were performed in triplicate.

### 2.9. Quantification of the IGFBP-6 and IGF-2 Secretion by Enzyme-Linked Immunosorbent Assay (ELISA)

Human IGFBP-6 (RayBiotech®, Burlington, ON) and IGF-2 (ALPCO, Salem, NH) ELISA kits were used to measure the amount of IGFBP-6 and IGF-2 secreted into the media of different treatment conditions. Standards and samples were loaded into the wells and the immobilized antibody bound IGFBP-6 or IGF-2 present in the sample. The wells were washed, and biotinylated anti-human antibody was added. After washing, HRP-conjugated streptavidin was added; then a TMB substrate solution was used to develop a blue color in proportion to the amount of IGFBP-6 or IGF-2 bound. The Stop Solution changes the color from blue to yellow, and the intensity of the color was measured at 450 nm using Multiskan Ascent analysis software.

### 2.10. Aldehyde Dehydrogenase (ALDH) Activity

PMSC ALDH activity was assessed by flow cytometry. An Aldefluor™ kit (Stem Cell Technologies, Vancouver, BC) was used as per the manufacturer's protocol. 5 *μ*L of the activated Aldefluor reagent/mL was added, and the cells were incubated for 45 minutes. Cells were centrifuged for 5 minutes and resuspended in 500 *μ*L of ice-cold Aldefluor assay buffer. ALDH activity was measured using flow cytometry. Samples were run in triplicate.

### 2.11. Antibodies

For pluripotency markers, we used OCT4 antibody (N-19: sc-8628) (Santa Cruz Biotechnology, Dallas, TX), and SOX2 (2683-1) (Epitomics, Burlington, ON, CAN). For muscle differentiation markers, Pax3/7 (E-10:sc365613), MyoD (M-318: sc-760), myogenin (F5D: sc-12732), and myosin heavy chain (H-300: sc-20641) (Santa Cruz Biotechnology, Dallas, TX) were used. For loading control, pan-actin Ab-5 (Thermo Fisher Scientific, Fremont, CA) was used. For IGFBP-6, antibody (H-70: sc-13094) (Santa Cruz Biotechnology, Dallas, TX) was used. The secondary antibodies used for immunoblotting were goat anti-rabbit (number 170-6515) or anti-mouse (number 170-6516) HRP-conjugated antibodies (Bio-Rad, Hercules, CA), or donkey anti-goat antibody (Santa Cruz Biotechnology, Dallas, TX). The secondary antibodies used for immunocytochemistry were green-Alexa 488 or red-Alexa 568 (Invitrogen, Mississauga, ON).

### 2.12. Statistical Analysis

All experiments were run in triplicates, and the specific protein levels were quantified and normalized for loading with the level of pan-actin in each lane. GraphPad Prism Software 5.0 was used to generate all graphs and analyses. A two-way ANOVA followed by a Bonferroni's multiple comparison test or a one-way ANOVA followed by a Student *t*-test was used, and significant difference was considered when *P* < 0.05. Graphic representation values are presented as mean ± SEM (shown as variance bars).

## 3. Results

### 3.1. PMSCs Can Differentiate into Skeletal Muscle

**Figure 1:**
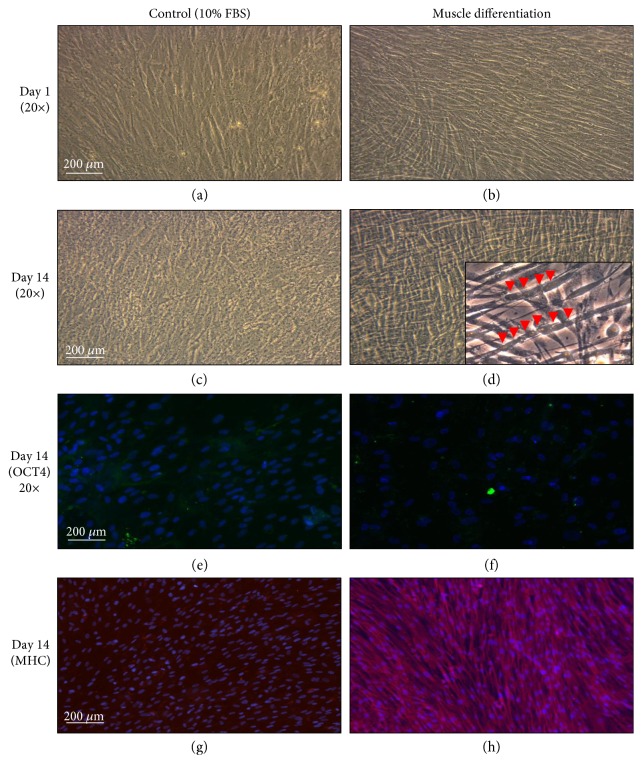
PMSCs cultured under muscle differentiation conditions showed muscle morphology with lower OCT4 and higher MHC at 14 days postdifferentiation. (a, b) Compared to cells cultured under standard conditions in 10% FBS, PMSCs grown in muscle differentiation media showed skeletal muscle morphology as early as day 1 postdifferentiation (20x). (c, d) At 14 days postdifferentiation, PMSCs grown in muscle differentiation media showed increased skeletal muscle fiber compaction and the formation of multinucleated fibers (20x). 40x magnification is shown in the bottom right corner. Red arrows indicate multinucleated muscle cells. (e–h) Cells grown in muscle differentiation media showed less OCT4 (green-Alexa 488, *λ*-488 nm) (20x) and more MHC immunoreactivity (red-Alexa 568, *λ*-568 nm), when compared to PMSCs in 10% FBS at 14 days postdifferentiation (10x). Nuclei were stained with Hoechst dye (blue, *λ* = 340 nm). Experiment was performed in triplicate.

To determine if PMSCs can differentiate into skeletal muscle, PMSCs were grown under muscle differentiation conditions for up to 14 days. Compared to PMSCs grown in nondifferentiating conditions (10% FBS), differentiated PMSCs showed muscle morphology as early as day 1 postdifferentiation (compaction and elongated appearance) (Figures 1(a) and 1(b)), and cells continued to differentiate forming multinucleated fibers at day 14 (Figures 1(c) and 1(d) and Supplementary Figure 3A and B). Associated with these morphological changes, pluripotency-associated marker (OCT4) immunoreactivity appeared low (Figures 1(e) and 1(f)), and muscle differentiation marker (MHC) immunoreactivity was high (Figures 1(g) and 1(h)) when compared to control cells (10% FBS). In addition, PMSCs under muscle differentiation conditions showed lower cell counts per field compared to undifferentiated controls (Supplementary Figure 3C).

**Figure 2:**
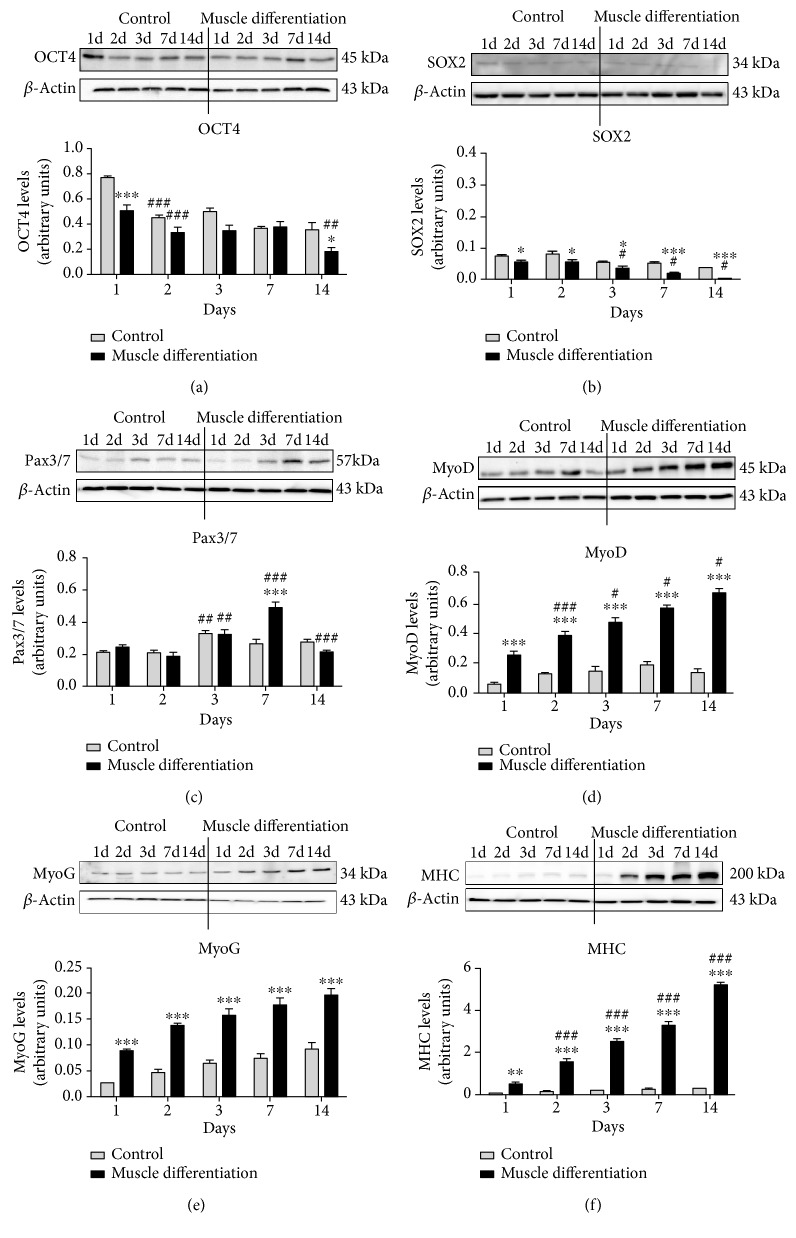
PMSCs cultured under muscle differentiation conditions increased levels of muscle markers (Pax3/7, MyoD, MyoG, and MHC) and decreased OCT4 and SOX2 levels using Western blots. Compared to PMSCs cultured under control conditions, (a) OCT4 was decreased at 1 and 14 days under muscle differentiation conditions. (b) SOX2 levels were decreased at each time point under muscle differentiation conditions. (c) Pax3/7 was increased at day 7 and decreased by day 14 under muscle differentiation conditions. (d) MyoD, (e) MyoG, and (f) MHC were increased at each time point under muscle differentiation conditions. Protein levels were quantified by densitometry and normalized to *β*-actin. Data is presented as the mean ± SEM of 3 independent experiments. Two-way ANOVA with Bonferroni's multiple comparison test was performed to determine ^∗^*P* < 0.05, ^∗∗^*P* < 0.01, and ^∗∗∗^*P* < 0.001 comparing control to muscle differentiation conditions, or ^#^*P* < 0.05, ^##^*P* < 0.01, and ^###^*P* < 0.001 comparing the same treatment over time.

Under muscle differentiation conditions, PMSCs decreased pluripotency-associated protein levels of OCT4 and SOX2. OCT4 levels were reduced at day 1 compared to control with further decrease at 14 days postdifferentiation (Figure 2(a)). In addition, SOX2 levels were lowered and nearly diminished by day 14 in cells under muscle differentiation conditions compared to control (Figure 2(b)). Muscle commitment marker Pax3/7 was increased at day 7, followed by a decrease at day 14 in PMSCs under muscle differentiation conditions compared to control (Figure 2(c)), suggesting that PMSCs under muscle differentiation conditions are committed to the muscle lineage and are proceeding to muscle differentiation. This was confirmed by the protein levels of muscle markers (MyoD, MyoG, and MHC) that increased significantly over time under muscle differentiation conditions (Figures 2(d), 2(e), and 2(f)). Collectively, these findings indicate that PMSCs differentiate into skeletal muscle under appropriate culture conditions, and this cell differentiation model could be consistently used to study muscle development *in vitro*.

**Figure 3:**
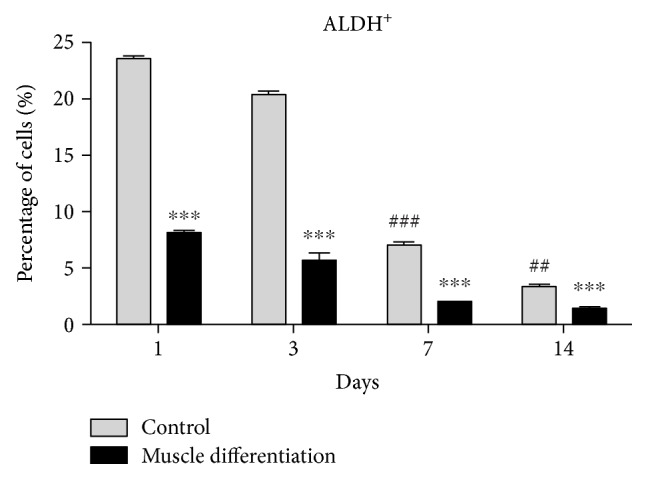
PMSCs cultured under skeletal muscle differentiation conditions showed a decreased frequency of cells with high ALDH activity. Compared to PMSCs cultured under control conditions, PMSCs cultured under differentiated conditions showed significantly decreased frequency of cells with high ALDH activity. Even under control culture conditions, PMSCs showed diminished ALDH-activity over time. Data is presented as the mean ± SEM of 3 independent experiments. Two-way ANOVA with Bonferroni's multiple comparison test was performed to determine ^∗∗∗^*P* < 0.001 comparing control to muscle differentiation conditions, or ^##^*P* < 0.01 and ^###^*P* < 0.001 comparing the same treatment over time.

We used the Aldefluor assay to determine the frequency of primitive progenitor cells with high ALDH activity. In this context, high ALDH activity is a conserved characteristic of proliferative progenitor cells of multiple lineages [25, 26]. As differentiation occurs towards a more mature cellular phenotype, ALDH activity is reduced. Compared to PMSCs grown under nondifferentiation conditions, there was a decrease in the frequency of cells with high ALDH activity (ALDH^+^ cells) under muscle differentiation conditions at days 1 to 14 (Figure 3 and Supplementary Figure 4). Moreover, ALDH activity was also decreased over time when cultured under control conditions (10% FBS) (Figure 3). These findings suggested that PMSCs comprised of a heterogeneous population that slowly differentiated during maintenance in standard culture conditions and PMSCs stimulated to differentiate into skeletal muscle immediately decreased ALDH activity at earlier time points.

### 3.2. PMSCs Express IGFBP-6 during Myogenic Differentiation

**Figure 4:**
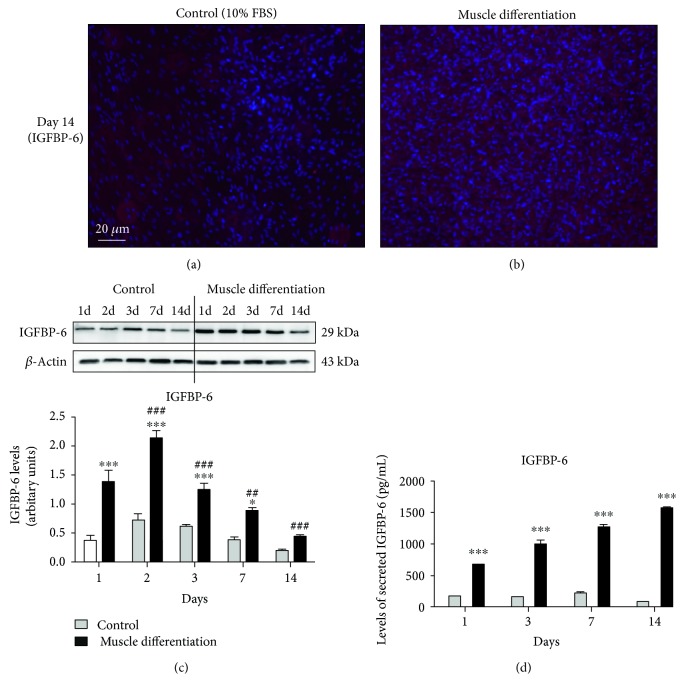
PMSCs cultured under skeletal muscle differentiation conditions showed increased IGFBP-6 expression and secretion. (a, b) PMSCs cultured under muscle differentiation conditions showed higher IGFBP-6 staining (red-Alexa, *λ*-568 nm) when compared to PMSCs under control conditions (10% FBS) at 14 days postdifferentiation. Nuclei were stained with Hoechst dye (blue, *λ* = 340 nm). (c) Using Western blots, IGFBP-6 protein levels in PMSCs cultured under differentiation conditions were increased at each time point compared to control conditions. Under muscle differentiation conditions, IGFBP-6 levels peaked at 2 days postdifferentiation and gradually decreased from days 3 to 14. Protein levels were quantified by densitometry and normalized to *β*-actin. (d) Using ELISA, IGFBP-6 and (e) IGF-2 secretion into the media was increased under muscle differentiation conditions compared to control conditions. Data is presented as the mean ± SEM of 3 independent experiments. Two-way ANOVA with Bonferroni's multiple comparison test was performed to determine ^∗^*P* < 0.05, and ^∗∗∗^*P* < 0.001 comparing control to muscle differentiation conditions, or ^##^*P* < 0.01 and ^###^*P* < 0.001 comparing the same treatment over time.

PMSCs under skeletal muscle differentiation conditions were investigated if they expressed IGFBP-6. Using immunocytochemistry, PMSCs cultured under differentiation conditions showed high intracellular IGFBP-6 immunoreactivity compared to PMSCs cultured under control conditions (Figures 4(a) and 4(b)). Using immunoblotting at multiple time points, following day 2 of differentiation, IGFBP-6 levels gradually decreased in PMSCs cultured under differentiation conditions but remained higher than time-matched controls (Figure 4(c)). Using ELISA detection in PMSC-conditioned media, there was an increase in the levels of IGFBP-6 secreted into the media, confirming that developing muscle cells express IGFBP-6 which is actively secreted into the extracellular space (Figure 4(d)). Therefore, the synthesis of IGFBP-6 increased as the cells became more differentiated towards the muscle lineage.

### 3.3. IGFBP-6 Affects Multipotency of the Developing Muscle Cells from PMSCs before Muscle Commitment

**Figure 5:**
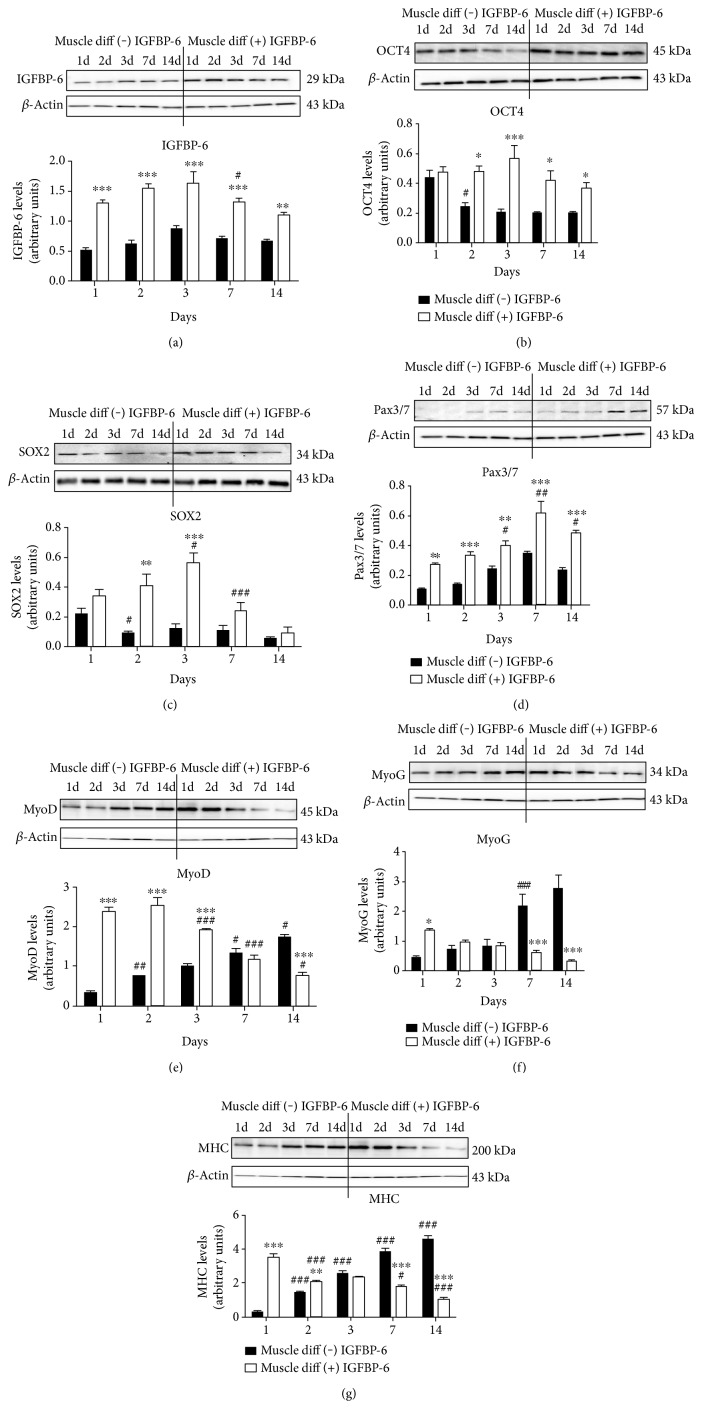
PMSCs treated with IGFBP-6 increased pluripotency-associated and muscle differentiation markers. IGFBP-6, OCT4, SOX2, Pax3/7, MyoD, MyoG, and MHC protein levels were quantified within PMSCs grown in muscle differentiation media with or without IGFBP-6 (375 ng/mL) supplementation using Western blots. (a) IGFBP-6 treatment increased IGFBP-6 levels as compared to PMSCs grown in muscle differentiation media only. IGFBP-6 treatment also increased pluripotency-associated markers (b) OCT4 and (c) SOX2 levels. (d) IGFBP-6 treatment increased muscle lineage commitment marker Pax3/7 at each time point. Muscle differentiation markers (e) MyoD, (f) MyoG, and (g) MHC levels increased with IGFBP-6 treatment at early time points (1–3 days) but showed reduced levels at later time points (7–14 days). Protein levels were quantified by densitometry and normalized to *β*-actin. Data is presented as the mean ± SEM of 3 independent experiments. Two-way ANOVA with Bonferroni's multiple comparison test was performed to determine ^∗^*P* < 0.05, ^∗∗^*P* < 0.01, and ^∗∗∗^*P* < 0.001 comparing treatments, or ^#^*P* < 0.05, ^##^*P* < 0.01, and ^###^*P* < 0.001 comparing the same treatment over time.

To test the effects of extracellular IGFBP-6 on developing muscle cells, recombinant human IGFBP-6 was added to the muscle differentiation media. Addition of extracellular IGFBP-6 into the culture media increased intracellular IGFBP-6 detection by Western blots, suggesting that recombinant human IGFBP-6 induced a positive feedback effect or was taken up by the differentiating cells (Figure 5(a)). Furthermore, stimulation in IGFBP-6 increased OCT4 and SOX2 levels concomitant to the increased IGFBP-6 levels (Figures 5(b) and 5(c)). Interestingly, IGFBP-6 supplementation also increased Pax3/7 levels suggesting enhanced PMSC commitment towards the skeletal muscle lineage (Figure 5(d)). The fact that these two events occurred simultaneously suggests that IGFBP-6 possibly had these effects on different population of cells in culture.

Finally, IGFBP-6 treatment increased the levels of muscle-specific markers, MyoD, MyoG, and MHC, at the earlier time points with a decline over time in the prolonged presence of increased extracellular IGFBP-6 compared to unsupplemented muscle differentiation conditions (Figures 5(e), 5(f), and 5(g)). Collectively, these data suggested that IGFBP-6 promoted PMSC commitment to the muscle lineage as an immediate effect but maintained pluripotency-associated markers and delayed muscle differentiation at later time points, as seen with the decreased protein level of muscle differentiation markers.

**Figure 6:**
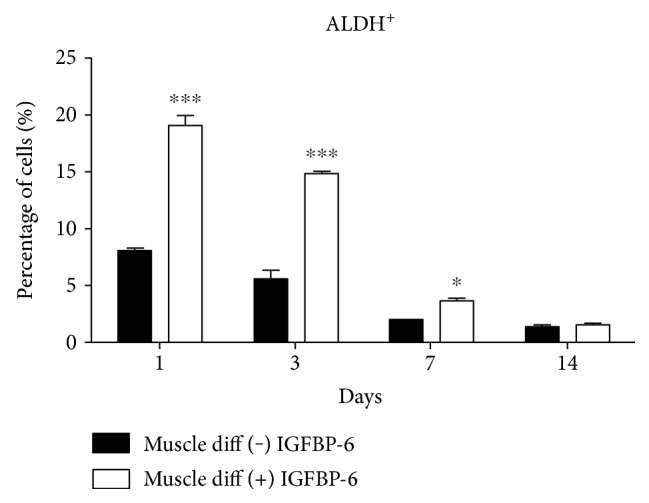
IGFBP-6 treatment increased the frequency of PMSCs with high ALDH activity. At days 1, 3, and 7, PMSCs treated with IGFBP-6 showed increased frequency of cells with high ALDH-activity. Data is presented as the mean ± SEM of 3 independent experiments. Two-way ANOVA with Bonferroni's multiple comparison test was performed to determine ^∗^*P* < 0.05 and ^∗∗∗^*P* < 0.001.

**Figure 7:**
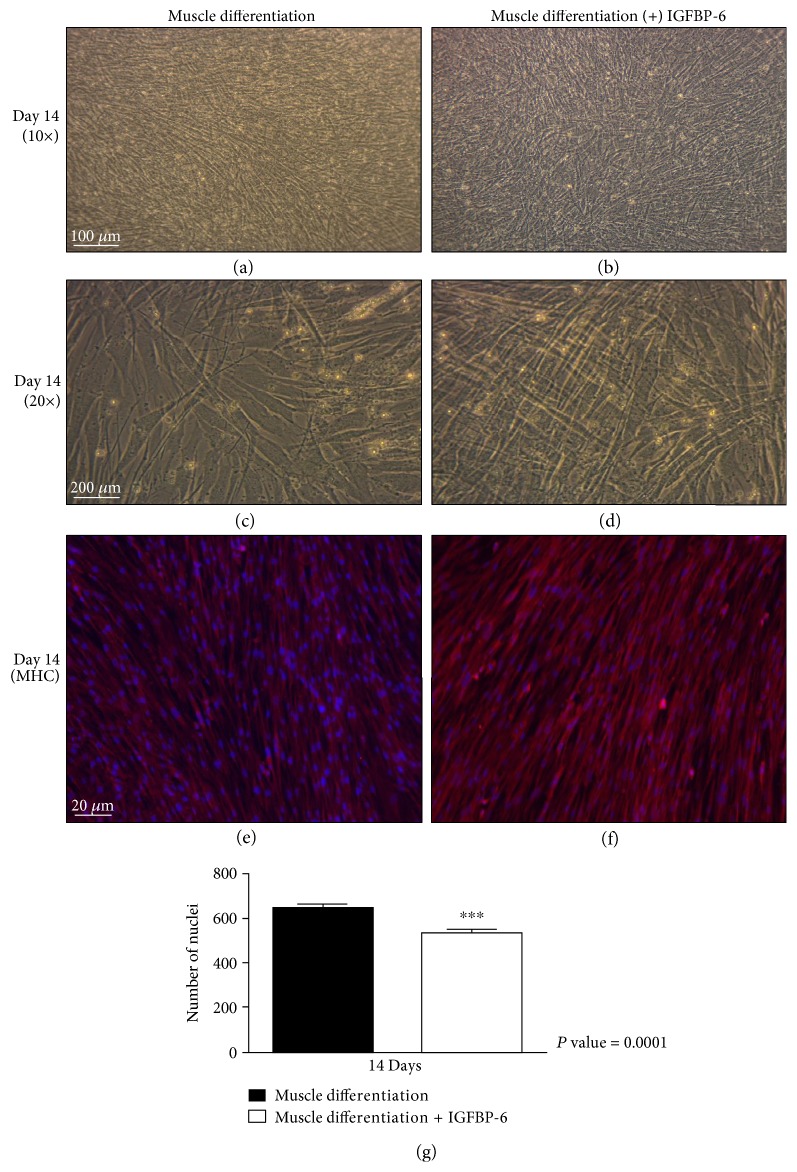
PMSCs under muscle differentiation conditions treated with IGFBP-6 showed more muscle compaction with lower cell count and similar immunofluorescence for MHC compared to cells under muscle differentiation alone at 14 days. Compared to PMSCs cultured under muscle differentiation alone, PMSCs treated with IGFBP-6 showed high cell compaction at day 14 (10x) (a, b) and (c, d) 20x. (e, f) Using immunocytochemistry, PMSCs were immunoreactive for MHC (red-Alexa, *λ*-568 nm), with no change in immunoreactivity with IGFBP-6 treatment. Nuclei were stained with Hoechst dye (blue, *λ* = 340 nm). Images are representative of 3 technical replicates. (g) PMSCs treated with IGFBP-6 had lower cell count at 14 days postdifferentiation compared to control PMSCs. Data is presented as the mean ± SEM of 15 different fields from 3 independent experiments. One-way ANOVA followed by Student's *t*-test, ^∗∗∗^*P* < 0.0001.

Due to the fact that both pluripotency-associated and differentiation markers increased by IGFBP-6 treatment in a time-dependent manner, we tested the cells for ALDH activity to determine the frequency of PMSCs that maintained high ALDH progenitor phenotype. PMSCs under muscle differentiation in the presence of IGFBP-6 increased ALDH activity compared to PMSCs under muscle differentiation alone at days 1 to 14 (Figure 6 and Supplementary Figure 5), suggesting that IGFBP-6 addition prolonged primitive progenitor phenotype in PMSCs cultured under muscle differentiation conditions. Further immunocytochemistry analyses at day 14 revealed that compared to unsupplemented conditions, PMSCs treated with IGFBP-6 showed more muscle compaction (Figures 7(a), 7(b), 7(c), and 7(d)). Moreover, MHC immunoreactivity appeared equivalent with or without IGFBP-6 supplementation (Figures 7(e) and 7(f)) with less number of cells (Figure 7(g)). These findings suggest that the increase in both pluripotency-associated and differentiation markers resulted from the impact of changing culture conditions (cellular environment) on a heterogeneous population of undifferentiated and differentiated cells.

### 3.4. Endogenous IGFBP-6 Is Required for the Differentiation of PMSCs to Skeletal Muscle

**Figure 8:**
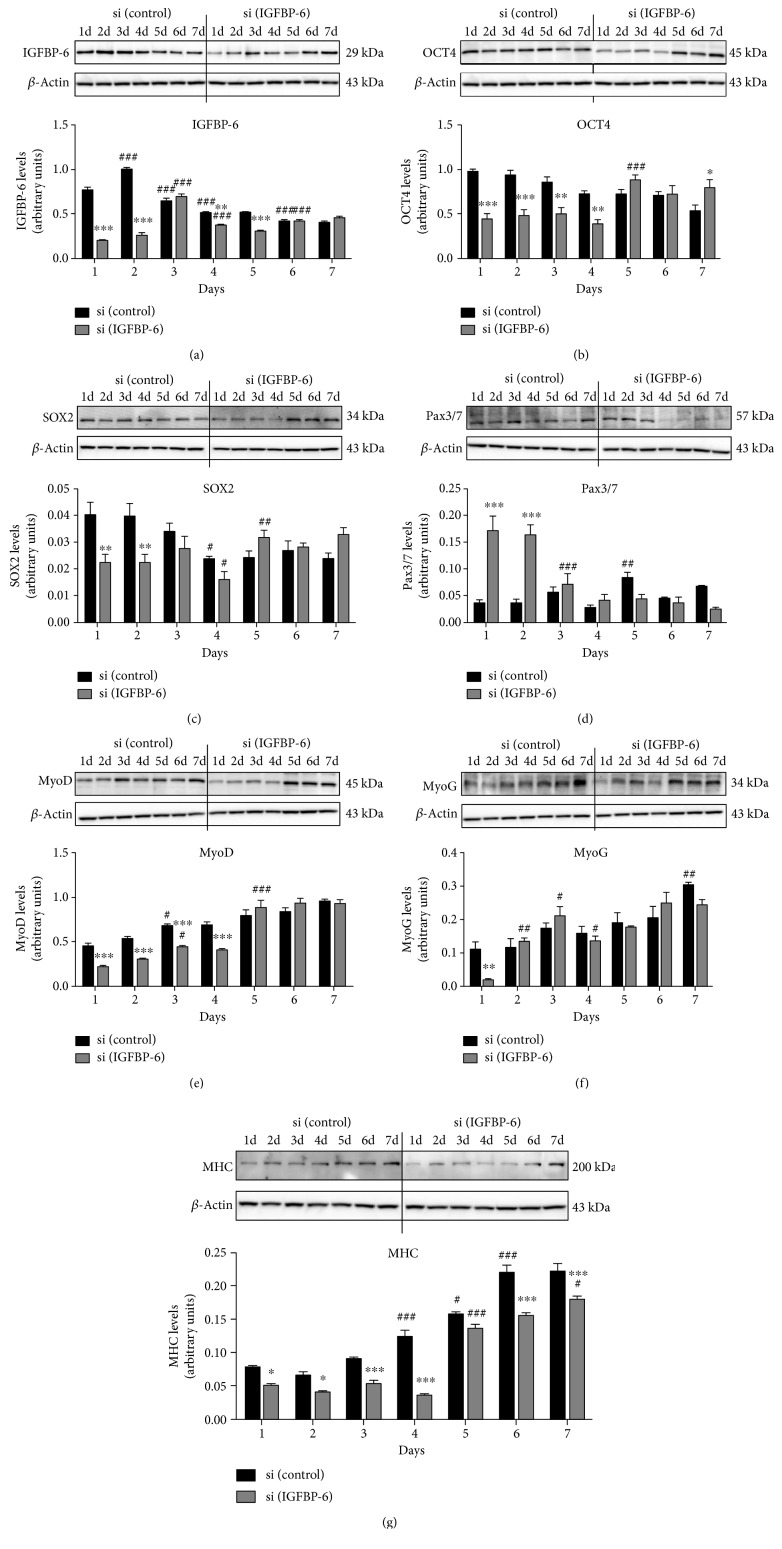
IGFBP-6 knockdown in PMSCs under muscle differentiation conditions reduced muscle markers protein levels. PMSCs were treated with siRNAs against IGFBP-6 or scrambled siRNA control every 3 days in muscle differentiation media. Using Western blots, (a) IGFBP-6 levels were significantly decreased at 1 and 2 days after siRNA treatment but recovered to control levels at day 3. When IGFBP-6 siRNA was reintroduced at day 3, there was a significant decrease for up to 5 days which returned to control levels at day 6. IGFBP-6 siRNA treatment decreased (b) OCT4 and (c) SOX2 levels at the early time points then returned to control levels at day 5. In contrast, muscle cell commitment marker (d) Pax3/7 was increased at days 1 and 2 when IGFBP-6 was knocked down. Muscle differentiation markers: (e) MyoD, (f) MyoG, and (g) MHC levels were decreased at early time points but recovered to control levels by day 5. Protein levels were quantified by densitometry and normalized to *β*-actin. Data is presented as the mean ± SEM of 3 independent experiments. Two-way ANOVA with Bonferroni's multiple comparison test was performed to determine ^∗^*P* < 0.05, ^∗∗^*P* < 0.01, and ^∗∗∗^*P* < 0.001 comparing siRNA treatments, or ^#^*P* < 0.05, ^##^*P* < 0.01, and ^###^*P* < 0.001 comparing the same treatment over time.

To evaluate the effects of IGFBP-6 silencing on pluripotency-associated and muscle differentiation markers in PMSCs, IGFBP-6 knockdown by siRNA was used during muscle differentiation over 7 days. As predicted, PMSC expression of IGFBP-6 was decreased for 1-2 days after IGFBP-6 knockdown compared to scrambled siRNA control. However, IGFBP-6 levels were equivalent to scrambled controls by day 3. Readministration of IGFBP-6 siRNA at day 3 prolonged IGFBP-6 reduction, but IGFBP-6 returned to control levels by day 6 (Figure 8(a)). These findings suggest that differentiating PMSCs have a high capacity to express IGFBP-6 and overcame siRNA knockdown within 3 days in culture. Alongside IGFBP-6 knockdown, we observed a reduction in pluripotency-associated markers for OCT4 (Figure 8(b)) and SOX2 (Figure 8(c)) concomitant with reduced IGFBP-6 levels, suggesting that IGFBP-6 may be important for maintaining potency which needs to be further investigated. Concomitantly, there was an increase in muscle commitment marker Pax3/7 that was reduced by day 3 (Figure 8(d)). Similarly, levels of the muscle lineage differentiation markers MyoD, MyoG, and MHC were all decreased at early time points after IGFBP-6 knockdown (Figures 8(e), 8(f), and 8(g)). Increased protein levels of muscle commitment marker and reduced levels of muscle differentiation markers suggest that endogenous IGFBP-6 knockdown initiated PMSCs commitment to the muscle lineage but delayed muscle differentiation.

**Figure 9:**
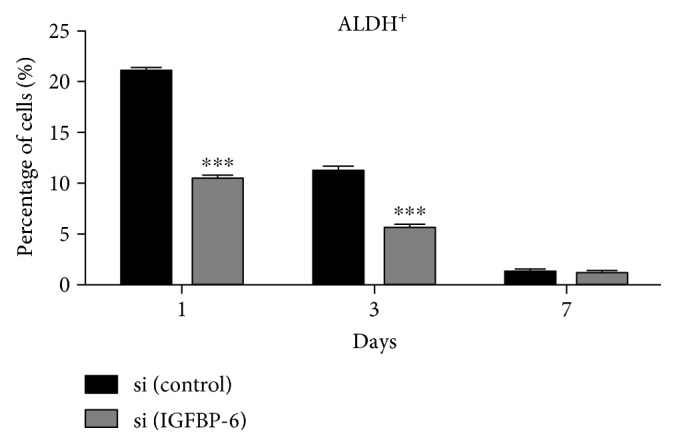
IGFBP-6 siRNA in PMSCs cultured under muscle differentiation conditions decreased the frequency of cells with high ALDH activity. PMSCs treated with IGFBP-6 siRNA showed significantly reduced frequency of cells with high ALDH activity. Data is presented as the mean ± SEM of 3 independent experiments. Two-way ANOVA with Bonferroni's multiple comparison test was performed to determine ^∗∗∗^*P* < 0.001.

**Figure 10:**
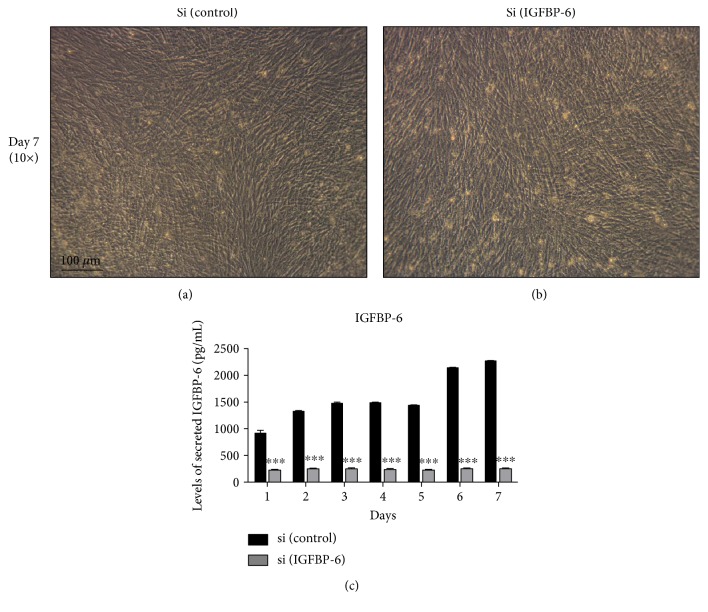
IGFBP-6 siRNA treatment maintained PMSCs cell morphology and inhibited IGFBP-6 secretion. PMSC skeletal muscle morphology was maintained for 7 days under muscle lineage differentiation conditions with (a) scrambled siRNA or (b) IGFBP-6 siRNA treatment. (c) Using ELISA, IGFBP-6 secretion was decreased at each time point with IGFBP-6 siRNA treatment that was applied every 3 days. Data is presented as the mean ± SEM of 3 independent experiments. Two-way ANOVA with Bonferroni's multiple comparison test was performed to determine ^∗∗∗^*P* < 0.001. ELISA sensitivity: highest amount detectable 60,000 pg/mL; lowest amount detectable 82.3 pg/mL. Standard curve *R*^2^ = 0.97.

As both pluripotency-associated and differentiation markers were decreased with IGFBP-6 silencing, ALDH activity was determined. Silencing of endogenous IGFBP-6 expression in PMSCs, decreased ALDH activity compared to control (scrambled siRNA) in a time-dependent manner (Figure 9 and Supplementary Figure 6). However, there was no change in cell morphology with IGFBP-6 silencing (Figure 10(a)) when compared to the control (Figure 10(b)) at day 7 postdifferentiation. On the other hand, IGFBP-6 knockdown decreased IGFBP-6 production and secretion as IGFBP-6 levels were reduced in PMSC-conditioned media at all time points as measured by ELISA (Figure 10(c)).

## 4. Discussion

Stem cell research has progressed in recent years, and the promise of using stem cells in tissue regeneration and cellular therapies are closer to becoming a reality in the clinics [27, 28]. However, before they can be used reliably and safely in regenerative medicine, it is essential to understand how factors within the stem cell microenvironment influence lineage commitment and differentiation as stem cell fate is altered by the culture conditions *in vitro* [29]. In addition, most current cellular therapies are expected to utilize pluripotent or multipotent stem cells that are already poised to generate into a desired lineage of committed progenitor cells by culturing them under specific culture conditions prior to therapy. Congenital muscular dystrophies represent a potential genetic disorder that may be amenable to cellular therapies due to accessibility and possible incorporation of new functional skeletal muscle cells into diseased tissues after transplantation [30, 31]. The results from this study are the first to provide insight on how IGFBP-6 can be used to modulate muscle lineage commitment and differentiation from readily available PMSCs *in vitro.*

To be able to use stem cells to treat Duchenne muscular dystrophy and to be approved for clinical trials, cells need to be from a readily available source, maintain the muscle differentiated state, avoid immune rejection by the host, avoid tumorigenesis, and can be easily injected. Human placental mesenchymal stem cells achieve these criteria.

The human placenta is usually discarded tissue after birth and represents a rich source of adult mesenchymal stem cells for the development of tissue regeneration therapies [2, 3, 32]. PMSCs have greater cell expansion and passage number *in vitro* than mesenchymal stem cells isolated from bone marrow [1]. They also demonstrate lower tumorigenicity [33] and higher immunotolerance capacity to reduce the possibility of triggering an immune response [34]. Thus, placental stem cells could provide an ethical and readily available source of stem cells for future experimental and clinical applications.

The IGF family plays a central role in muscle development, differentiation, growth, and regeneration [20–22, 35, 36]. In Duchenne muscular dystrophy, IGF-1 activates muscle growth and hypertrophy and appears to improve the loss of muscle mass [37]. IGFBPs are the carriers for IGFs in the circulation [7], protecting them from degradation [12, 38] and delivering them to specific tissues and thus modulate the biological actions of IGFs. Also, IGFBPs increase the half-life of IGF peptides in the circulation and control their access to the IGF-1R, thus playing an important role in IGF-regulated cell metabolism, development, and growth. In recent years, it has become apparent that the IGFBPs can be expressed and maintained within the cellular environment and have functions independent of IGFs [14]. Several IGF binding proteins have been shown to be important in myogenesis and are expressed in developing muscle cells. Ren et al. reported that in C2C12 myoblast cells and in primary skeletal muscle cells, IGFBP-5 acts in an IGF-dependent manner to promote myogenesis by binding to IGF-2 and promoting its interaction with the IGF-1R [39]. Knockdown of IGFBP-5 impaired myogenic differentiation by reducing myogenin, myosin heavy chain, and IGF-2 expression [39]. In L6E9 skeletal myoblasts, IGFBP-4 and IGFBP-6 were accumulated during myogenesis, with IGFBP-4, and not IGFBP6, inhibiting IGF-1 induced muscle differentiation [40]. These findings suggested the important role of IGFBPs in the muscle differentiation of both primary and cell lines of skeletal muscle lineage. Our study is the first to demonstrate the role of IGFBP-6, which is specific for the embryonic IGF, IGF-2, in muscle development using PMSCs.

The aim of this study was to characterize the effects of IGFBP-6 on the early differentiation stage before PMSCs commit to the muscle lineage. As shown, when PMSCs were cultured under muscle differentiation-specific conditions, they showed the capacity to differentiate into multinucleated muscle fibers and commit to the muscle lineage. The biological effects of IGFBP-6 on this differentiation process, as determined by pluripotency-associated markers (OCT4 and SOX2), muscle commitment (Pax3/7), and differentiation (MyoD, MyoG, and MHC), were significantly changed at the earlier time points. Thus, IGFBP-6 induced muscle differentiation and could potentially be used to guide skeletal muscle regeneration using stem cell therapy.

IGFBP-6 was highly expressed in developing muscle cells [41, 42]; however, its role in muscle development is unclear. Previous studies from our laboratory using human fetal tissues have demonstrated that IGFBP-6 mRNA was expressed abundantly in the skeletal muscle, heart, and skin and prevalent in the regions of active cellular division and differentiation, suggesting that the protein is synthesized in these tissues and has autocrine/paracrine actions in the developing cells [43]. In another study from our laboratory, we reported that IGFBP-6 mRNA was expressed in low abundance in the chorionic villi of placenta during the second and third trimesters [44], suggesting that this IGFBP-6 is expressed in specific population of cells in this tissue (e.g., mesenchymal stem cells) and/or that the expression is increased only when PMSCs are induced to differentiate into a specific lineage such as skeletal muscle.

The findings in this current study using PMSCs suggest that stem cells in the developing myotome or MSCs in a developed muscle tissue express IGFBP-6 in significant levels during differentiation, indicating IGFBP-6 as an integral protein during muscle development. In fact, as muscle differentiation progressed *in vitro*, the intracellular IGFBP-6 decreased gradually due to the increased capacity to secrete IGFBP-6 into the culture medium, indicating multiple roles for IGFBP-6, both intracellular and extracellular in muscle development. Thus, IGFBP-6 activities may switch from intracellular IGF-independent actions to more paracrine IGF-dependent or IGF-independent actions as muscle differentiation occurs. Interestingly, the increase in extracellular IGFBP-6 by the addition of IGFBP-6 to the culture medium significantly increased cellular IGFBP-6 (intracellular or cell associated) with a concurrent increase in pluripotency-associated markers OCT4 and SOX2. The increase in intracellular IGFBP-6 suggests that IGFBP-6 was likely internalized or associated with the cell surface. A previous report from our laboratory demonstrated the intracellular actions of IGFBP-6 in the cytoplasm and nucleus of skeletal muscle cell line RD cells which is likely an IGF-independent actions of IGFBP-6 [19].

When extracellular IGFBP-6 was supplemented into PMSC cultured under muscle differentiation conditions, the muscle commitment marker Pax3/7 was increased at all time points of study, while other muscle differentiation markers increased only at the earlier time points. As the differentiation progressed, IGFBP-6 treatment inhibited complete myogenic differentiation as demonstrated by decreased muscle differentiation markers MyoD, MyoG, and MHC. These findings together with the higher OCT4 and SOX2 levels indicate that IGFBP-6 promotes the commitment of PMSCs towards the muscle lineage, while the prolonged presence delays the differentiation process. Moreover, increased IGFBP-6 in the MSC microenvironment is expected to reduce the bioavailability of IGF-2 due to its high affinity for the peptide, confirmed by IGF-2 ELISA (Supplementary Figure 7A). Thus, it is likely that the increased IGF-2 secretion by the differentiating muscle cells will have a biologic impact on muscle development which will be further investigated.

Knockdown of IGFBP-6 using siRNA decreased both intracellular and secreted IGFBP-6. This knockdown-mediated decrease in OCT4 and SOX2 supports the evidence that IGFBP-6 enhances pluripotency of PMSCs. In contrast, the significant early increase in Pax3/7 with IGFBP-6 silencing supports an earlier commitment towards the myogenic lineage. The increase in Pax3/7 could be due to the presence of a greater availability of extracellular IGF-2 (Supplementary Figure 7B), which is being recruited to the commitment process or could be due to actions independent of IGFBP-6. In contrast, the muscle differentiation markers (MyoD, MyoG, and MHC) were all reduced after IGFBP-6 knockdown, suggesting that IGFBP-6 is required for the muscle differentiation process. Therefore, IGFBP-6 supports PMSC multipotency and its loss leads to an early commitment towards the myogenic lineage but delayed differentiation.

Studies in various cell lines have shown mostly an inhibitory action of IGFBP-6 mainly via IGF-2-dependent actions. In L6A1 myoblast, IGFBP-6 inhibited muscle differentiation induced by IGF-2 but not IGF-1 [45]. Previous reports on the effects of IGFs on muscle differentiation were using mouse cell lines [46–49]; thus, our study is one of the first to show the effects of IGFBP-6 on human mesenchymal stem cell differentiation into skeletal muscle *in vitro*.

Overall, we have demonstrated in this study that IGFBP-6 has both endogenous and exogenous actions that can promote or inhibit PMSC multipotency or differentiation. Exogenous IGFBP-6 exposure facilitates muscle lineage commitment while a prolonged exposure can inhibit late stage differentiation. Therefore, endogenous IGFBP-6 is required for maintaining multipotency and delaying commitment and enhancing late stage differentiation.

**Figure 11:**
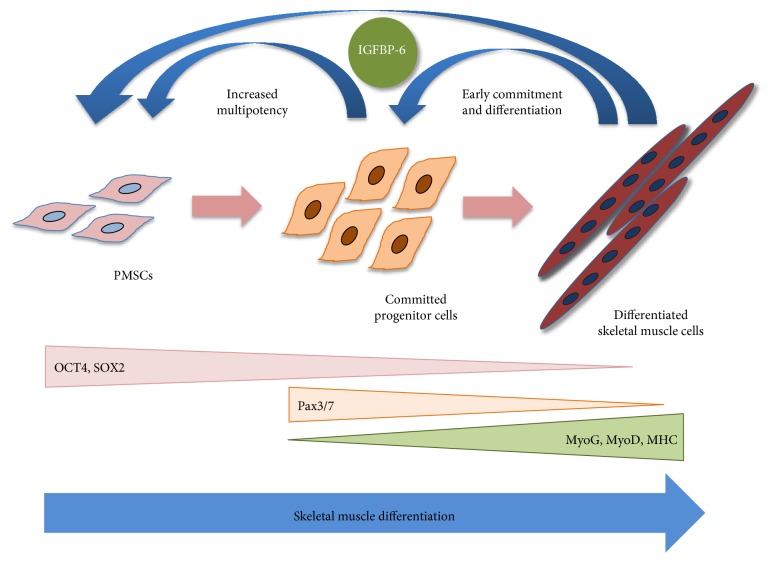
Model of IGFBP-6 functions on PMSC differentiation into skeletal muscle. PMSCs under normal growth conditions (10% FBS) express high levels of pluripotency-associated markers OCT4 and SOX2. As these cells commit towards the skeletal muscle lineage, increased IGFBP-6 correlated with increased Pax3/7 that decreased as differentiation markers (MyoG, MyoD, and MHC) were increased. Both committed and differentiated muscle cells continued to express and secrete IGFBP-6. As IGFBP-6 increased, there was an increase in multipotency markers, as well as, an earlier commitment and differentiation towards the muscle lineage. Thus, IGFBP-6 was required for maintaining multipotency and enhancing muscle commitment and differentiation.

In conclusion, PMSCs are able to differentiate into skeletal muscle cells under appropriate environment or niche conditions and that this process is enhanced by the increase in extracellular IGFBP-6 and delayed by silencing the endogenous expression as evident by alterations in both pluripotent and muscle differentiation markers (Figure 11). A balance between endogenous and exogenous levels of IGFBP-6 is required for the complete muscle differentiation process, and since IGFBP-6 has intracellular as well as extracellular effects, whether the response occur dependent or independent of IGFs (particularly IGF-2) will be further delineated.

## Supplementary Material

Supplementary Figure 1: PMSC Isolation from 15 weeks preterm placenta. (A) The dissected villous tissue was digested enzymatically and cells were separated using a discontinuous Percoll gradient. Five cell fractions were typically obtained corresponding to five different densities and cells were isolated from layers 3, 4, and 5. (B) Phase contrast images of the isolated PMSCs, from all three layers, grown in culture after 4 weeks. (C) PMSCs from passage 4 of all three layers were positive for CD73 and CD105 (>98%), and were negative for CD117 (<1%) (measured by flow cytometry). Flow cytometry histograms are representative of all 3 layers from 3 placental tissue as they showed the same results. Supplementary Figure 2: IGFBP-6 levels in response to IGFBP-6 supplementation in PMSCs under skeletal muscle differentiation conditions. (A) PMSCs cultured under muscle differentiation conditions showed increased IGFBP-6 protein levels, using western blots, in response to different doses of recombinant human IGFBP-6 protein supplementation with 375 ng/mL and 450 ng/mL having the highest band intensity. (B) IGFBP-6 secretion into the media was increased with the supplementation of recombinant human IGFBP-6 protein (375 ng/mL) that reduced by time and was lower compared to control at day 3. Data is presented as the mean ± SEM of 3 independent experiments. Two-way ANOVA with Bonferroni's multiple comparison test was performed to determine ^∗∗∗^*P* < 0.001. Supplementary Figure 3: PMSCs cultured under muscle differentiation conditions showed the formation of multi-nucleated fibers and lower cell count compared to control. (A) At 14 days post-differentiation, PMSCs are immunoreactive for MHC (Red-Alexa 568, *λ*-568 nm) with cell alignment and multi-nucleated fiber formation (5X). Nuclei, were stained with Hoechst dye (blue, *λ*=340 nm). (B) PMSCs grown in muscle differentiation media showed multi-nucleated skeletal muscle fiber formation (40X). Black arrows indicate the multi-nucleated muscle. (C) PMSCs under muscle differentiation conditions showed lower cell count per field compared to control. Data is presented as the mean ± SEM of 15 different fields from 3 independent experiments. One-way ANOVA followed by a Student's *t*-test, ^∗∗^*P* < 0.01. Supplementary Figure 4: PMSCs cultured under skeletal muscle differentiation conditions showed a decreased frequency of cells with high ALDH-activity. Representative flow cytometry dot plots showing the frequency of PMSC with high ALDH-activity with Aldefluor and an inhibitor of ALDH (DEAB) or with ALDH alone when cultured under control (10% FBS) or muscle differentiation conditions at (A) day 1, (B) day 3, (C) day 7, (D) and day 14. Supplementary Figure 5: IGFBP-6 treatment increased the frequency of PMSCs with high ALDH-activity. Representative flow cytometry dot plots with Aldefluor and an inhibitor (DEAB) or with ALDH alone in PMSCs cultured under muscle differentiation conditions with or without IGFBP-6 addition at (A) day 1, (B) day 3, (C) day 7, (D) and day 14. Supplementary Figure 6: IGFBP-6 siRNA in PMSCs cultured under muscle differentiation conditions decreased the frequency of cells with high ALDH-activity. Representative flow cytometry dot plots with Aldefluor and an inhibitor of ALDH (DEAB) or with ALDH alone of PMSCs treated with IGFBP-6 siRNA at (A) day 1, (B) day 3, and (C) day 7 under muscle differentiation conditions. Supplementary Figure 7: IGF-2 secretion in PMSCs treated with IGFBP-6 or IGFBP-6 siRNA under muscle differentiation conditions. (A) IGF-2 levels secreted into the media were significantly decreased at each time point after IGFBP-6 addition compared the control. (B) After treatment with siRNA against IGFBP-6 compared to controls (scrambled siRNA), IGF-2 levels increased at the first 48 hours with siRNA treatment applied every 3 days. Data is presented as the mean ± SEM of 3 independent experiments. Two-way ANOVA with Bonferroni's multiple comparison test was performed to determine ^∗^*P* < 0.05, ^∗∗^*P* < 0.001.
