# Supplementary material for: Insulin-Like Growth Factor Binding Protein-6 Alters Skeletal Muscle Differentiation of Human Mesenchymal Stem Cells
Source: Stem Cells Int. 2017 Sep 14;2017:2348485. doi: 10.1155/2017/2348485 (PMC5618785; doi:10.1155/2017/2348485)

Supplementary Figures

Supplementary Figure 1: PMSC Isolation from 15 weeks preterm placenta. (A) The dissected villous tissue was digested enzymatically and cells were separated using a discontinuous Percoll gradient. Five cell fractions were typically obtained corresponding to five different densities and cells were isolated from layers 3, 4, and 5. **(B)** Phase contrast images of the isolated PMSCs, from all three layers, grown in culture after 4 weeks. **(C)** PMSCs from passage 4 of all three layers were positive for CD73 and CD105 (>98%), and were negative for CD117 (<1%) (measured by flow cytometry). Flow cytometry histograms are representative of all 3 layers from 3 placental tissue as they showed the same results.

Supplementary Figure 2: IGFBP-6 levels in response to IGFBP-6 supplementation in PMSCs under skeletal muscle differentiation conditions. (A) PMSCs cultured under muscle differentiation conditions showed increased IGFBP-6 protein levels, using western blots, in response to different doses of recombinant human IGFBP-6 protein supplementation with 375 ng/mL and 450 ng/mL having the highest band intensity. **(B)** IGFBP-6 secretion into the media was increased with the supplementation of recombinant human IGFBP-6 protein (375 ng/mL) that reduced by time and was lower compared to control at day 3. Data is presented as the mean ± SEM of 3 independent experiments. Two-way ANOVA with Bonferroni's multiple comparison test was performed to determine ***P<0.001.

Supplementary Figure 3: PMSCs cultured under muscle differentiation conditions showed the formation of multi-nucleated fibers and lower cell count compared to control. (A) At 14

days post-differentiation, PMSCs are immunoreactive for MHC (Red-Alexa 568, λ-568 nm) with cell alignment and multi-nucleated fiber formation (5X). Nuclei, were stained with Hoechst dye (blue, λ=340 nm). **(B)** PMSCs grown in muscle differentiation media showed multi-nucleated skeletal muscle fiber formation (40X). Black arrows indicate the multi-nucleated muscle. **(C)** PMSCs under muscle differentiation conditions showed lower cell count per field compared to control. Data is presented as the mean ± SEM of 15 different fields from 3 independent experiments. One-way ANOVA followed by a Student's t-test, **P<0.01.

Supplementary Figure 4: PMSCs cultured under skeletal muscle differentiation conditions showed a decreased frequency of cells with high ALDH-activity. Representative flow cytometry dot plots showing the frequency of PMSC with high ALDH-activity with Aldefluor and an inhibitor of ALDH (DEAB) or with ALDH alone when cultured under control (10% FBS) or muscle differentiation conditions at **(A)** day 1, **(B)** day 3, **(C)** day 7, **(D)** and day 14.

Supplementary Figure 5: IGFBP-6 treatment increased the frequency of PMSCs with high ALDH-activity. Representative flow cytometry dot plots with Aldefluor and an inhibitor (DEAB) or with ALDH alone in PMSCs cultured under muscle differentiation conditions with or without IGFBP-6 addition at **(A)** day 1, **(B)** day 3, **(C)** day 7, **(D)** and day 14.

Supplementary Figure 6: IGFBP-6 siRNA in PMSCs cultured under muscle differentiation conditions decreased the frequency of cells with high ALDH-activity. Representative flow cytometry dot plots with Aldefluor and an inhibitor of ALDH (DEAB) or with ALDH alone of

PMSCs treated with IGFBP-6 siRNA at **(A)** day 1, **(B)** day 3, and **(C)** day 7 under muscle differentiation conditions.

Supplementary Figure 7: IGF-2 secretion in PMSCs treated with IGFBP-6 or IGFBP-6 siRNA under muscle differentiation conditions. (A) IGF-2 levels secreted into the media were significantly decreased at each time point after IGFBP-6 addition compared the control. **(B)** After treatment with siRNA against IGFBP-6 compared to controls (scrambled siRNA), IGF-2 levels increased at the first 48 hours with siRNA treatment applied every 3 days. Data is presented as the mean ± SEM of 3 independent experiments. Two-way ANOVA with Bonferroni's multiple comparison test was performed to determine $*P<0.05$, $**P<0.001$.

Supplementary Figure 1.

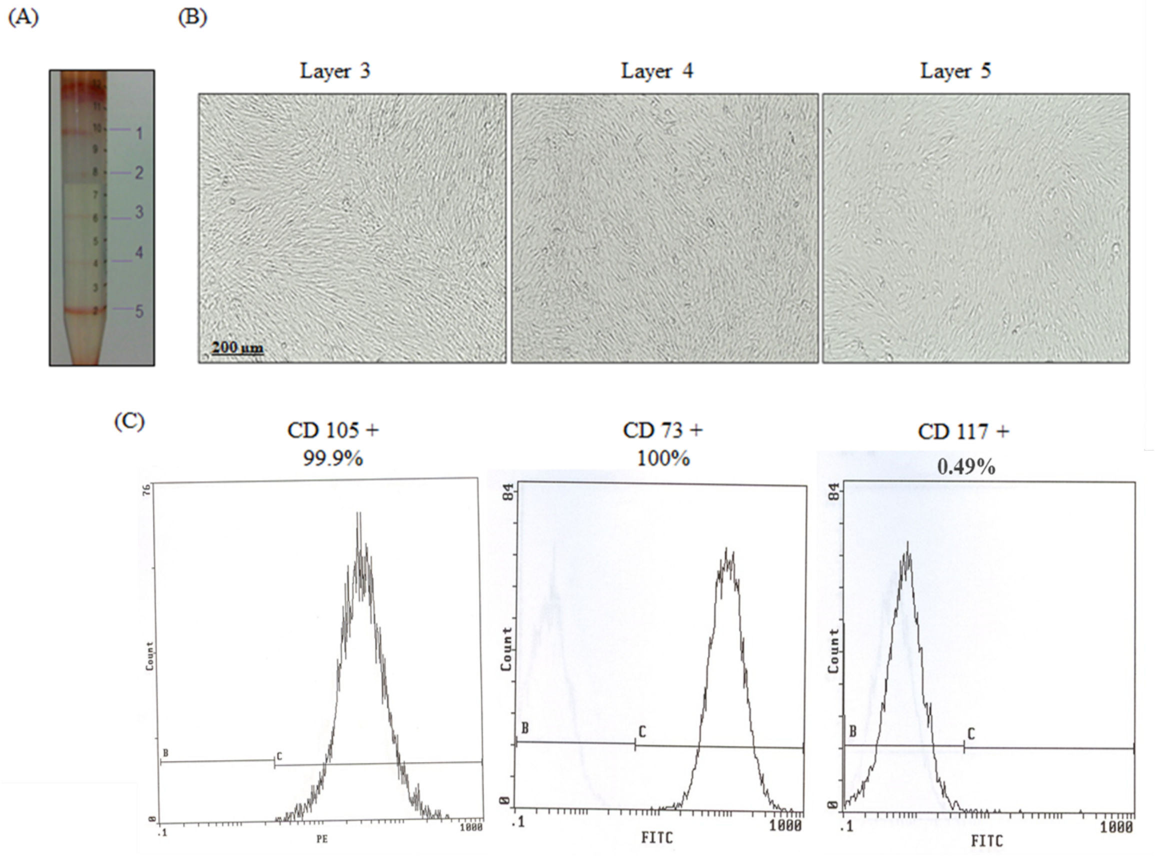

Supplementary Figure 2.

(A)

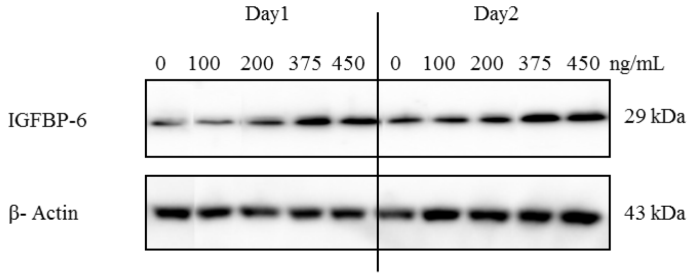

(B)

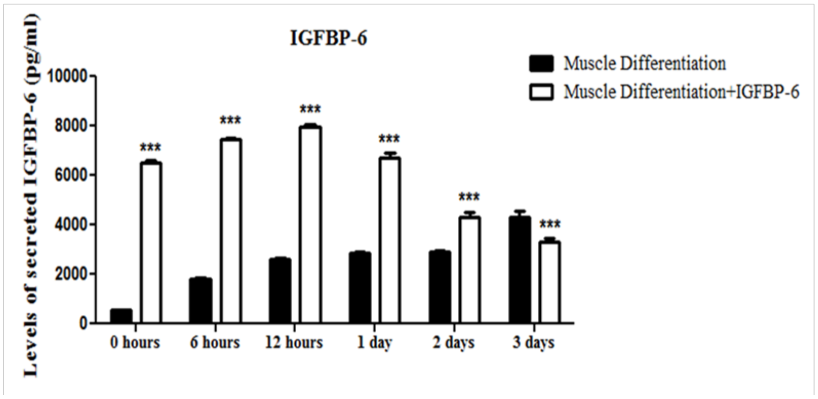

Supplementary Figure 3.

(A)

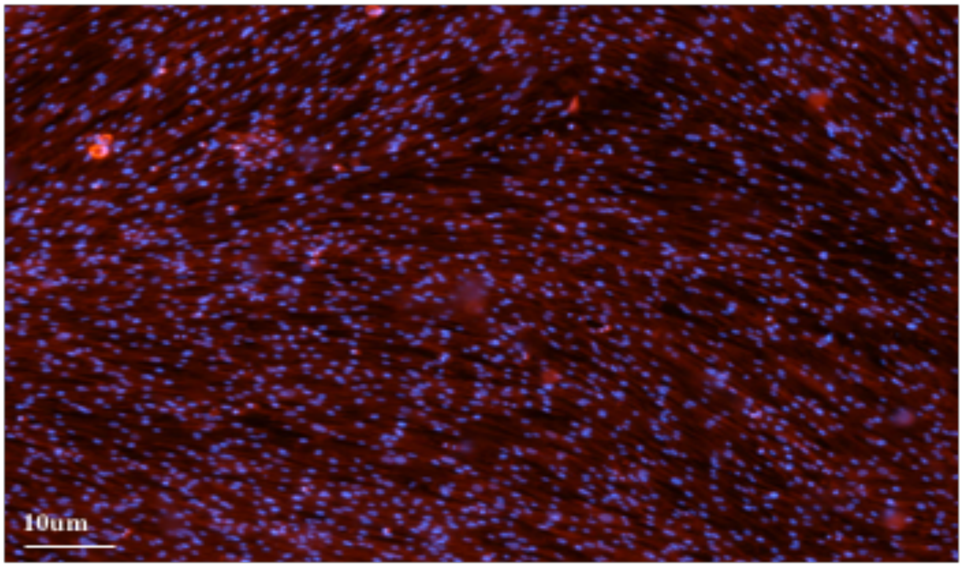

(B)

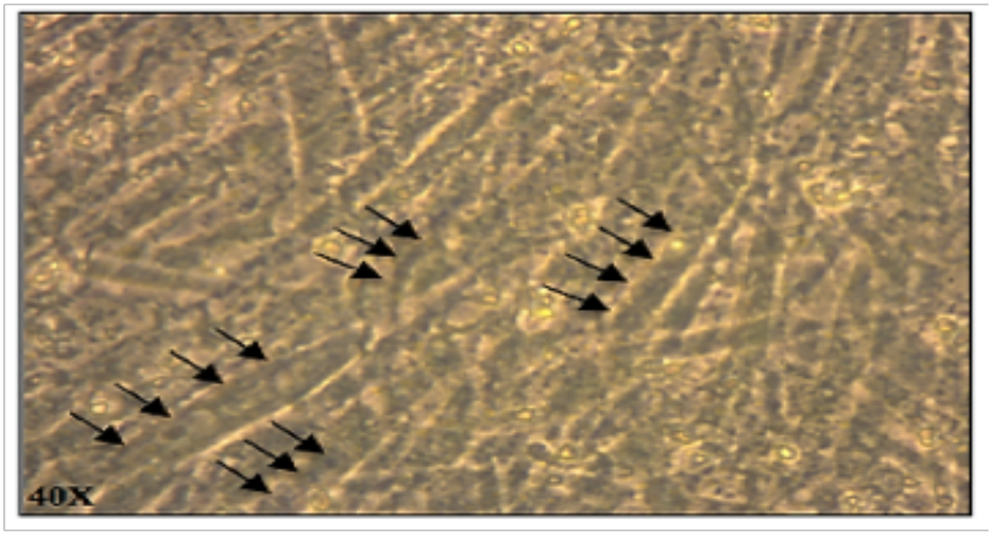

(C)

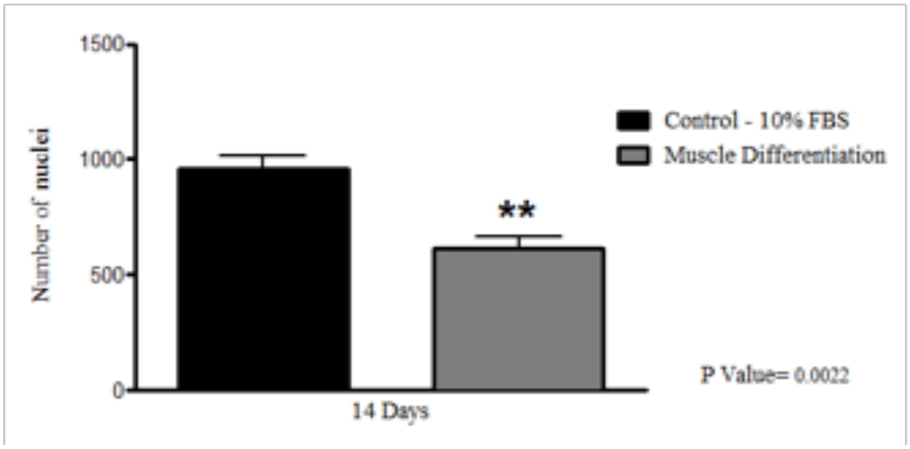

Supplementary Figure 4.

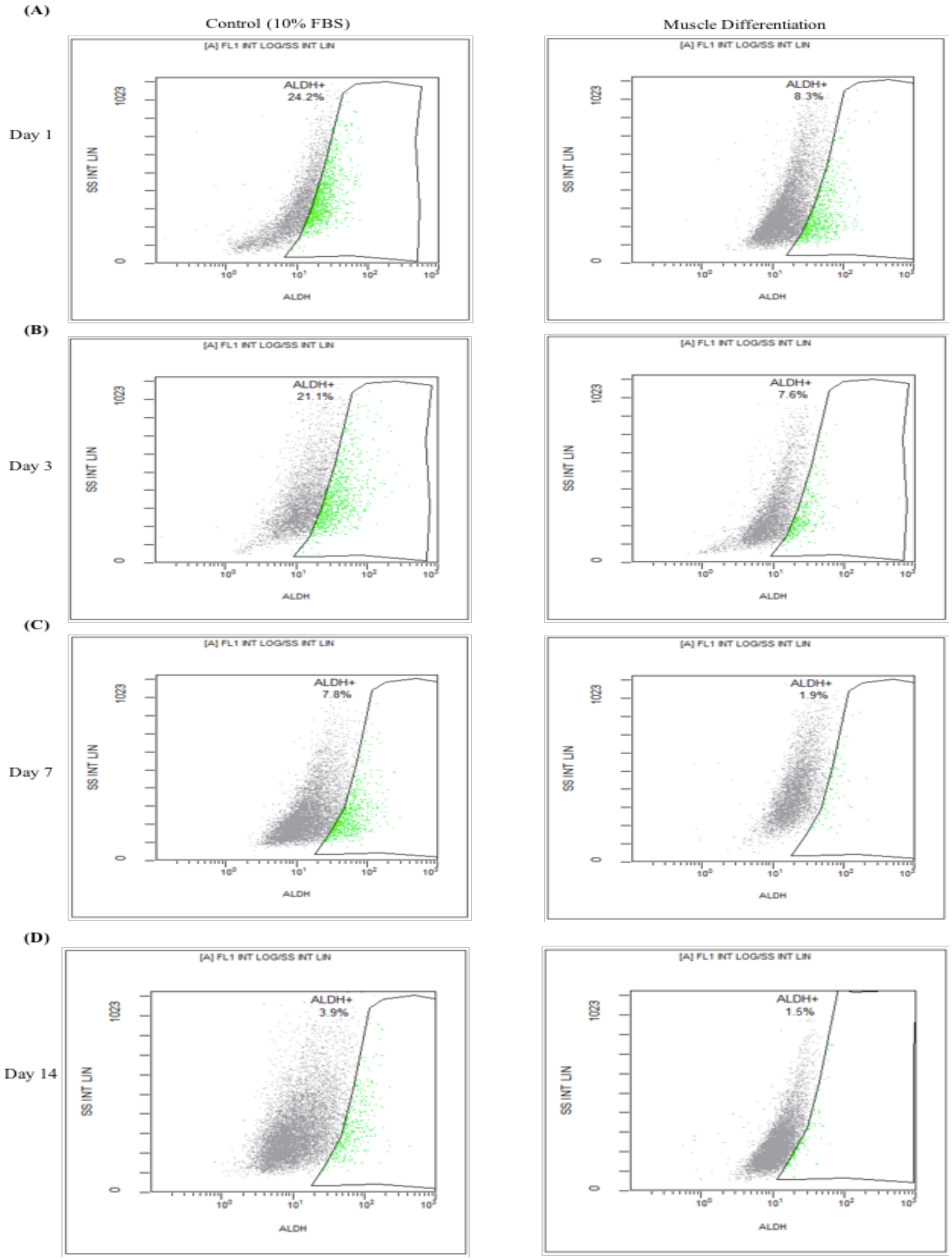

Supplementary Figure 5.

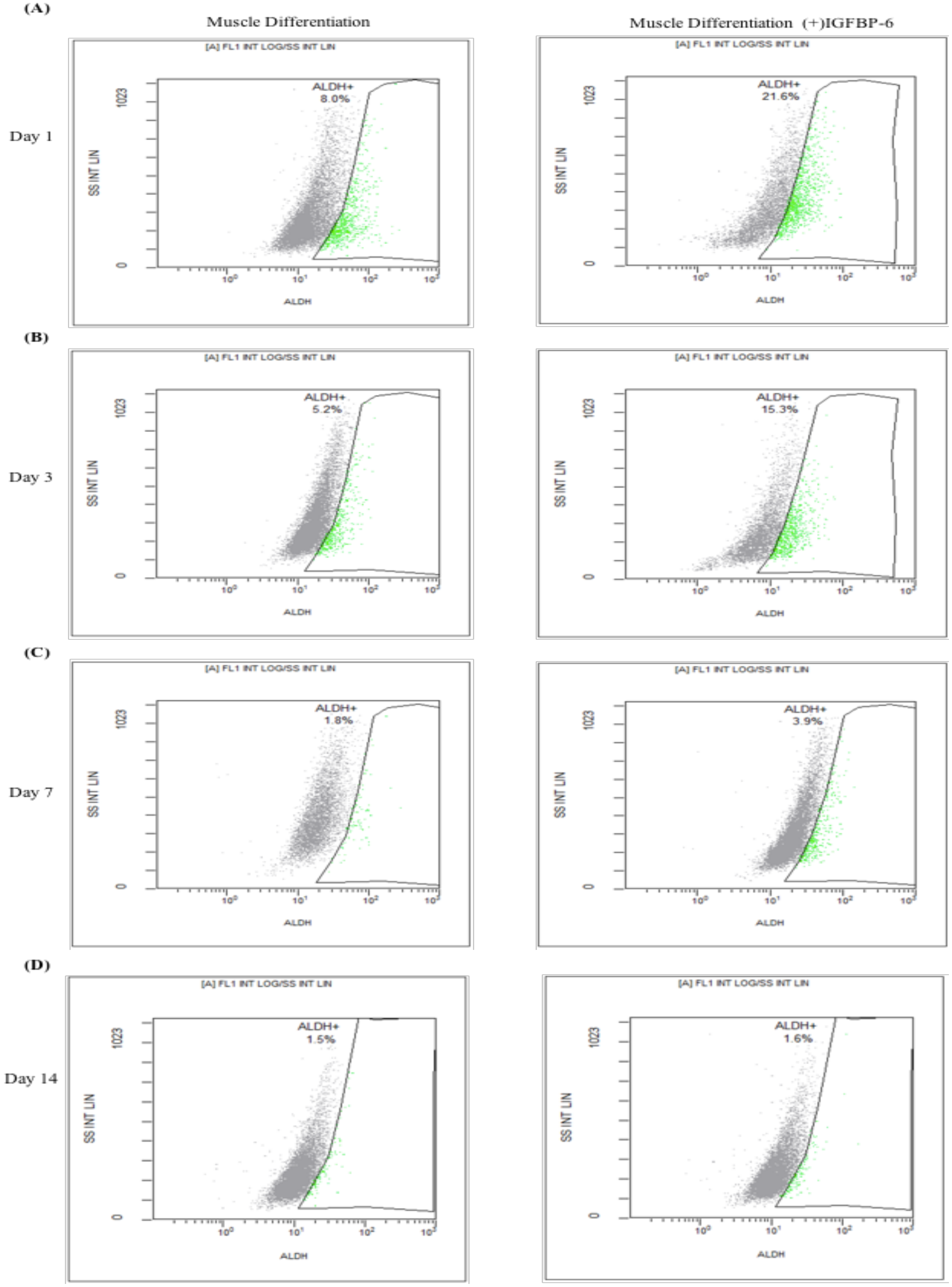

Supplementary Figure 6.

(A)

Si (Control) Si (IGFBP-6)

Day 1

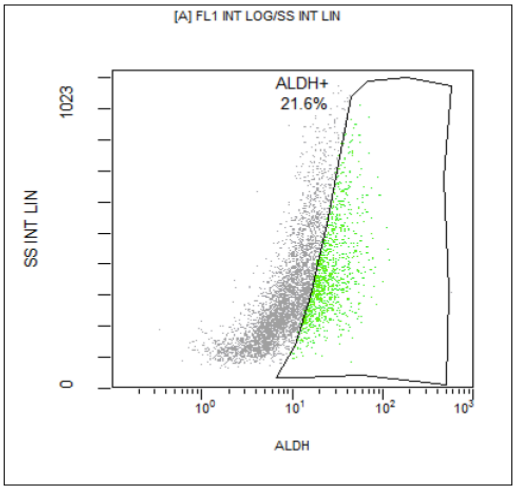

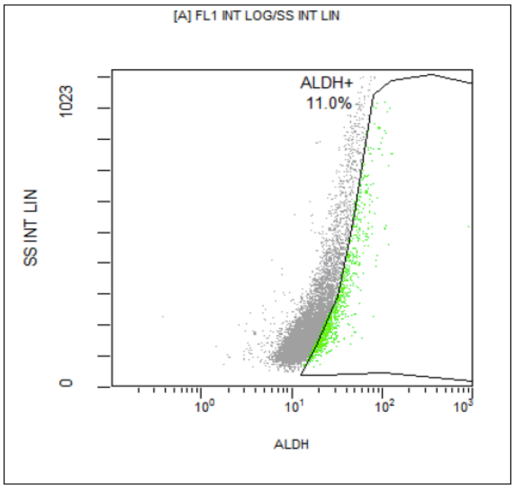

(B)

Day 3

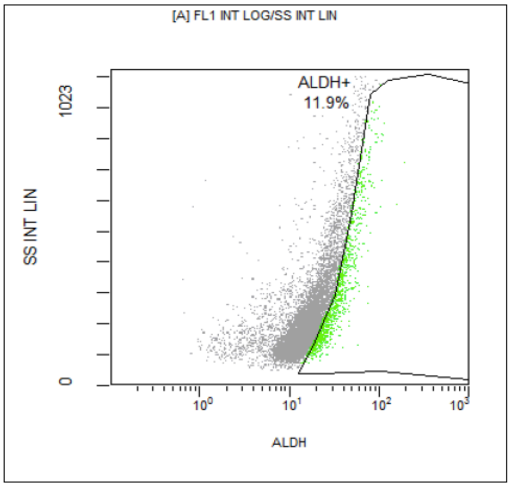

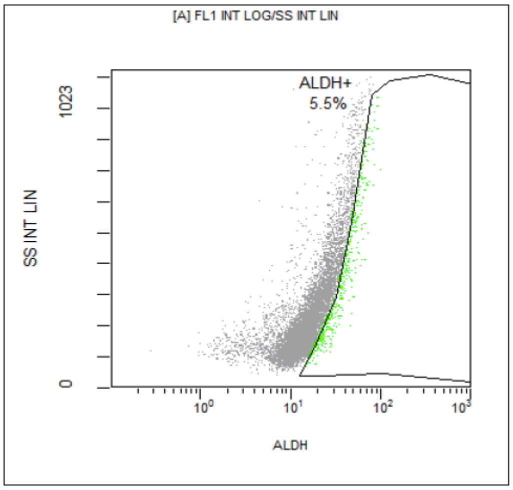

(C)

Day 7

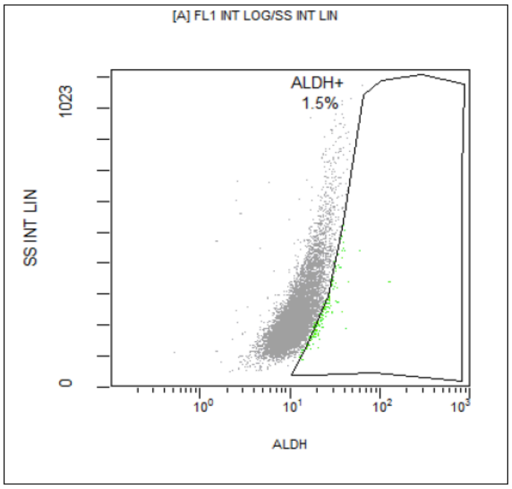

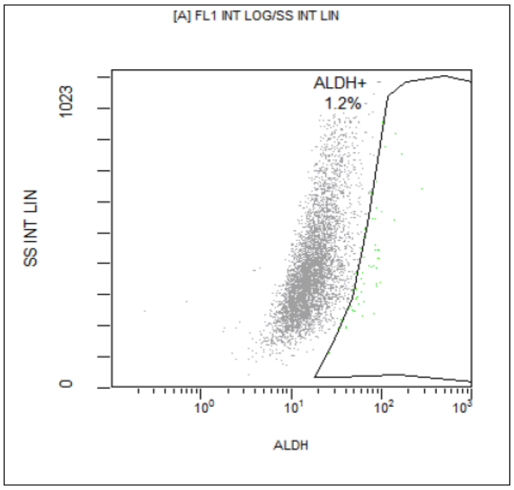

Supplementary Figure 7.

(A)

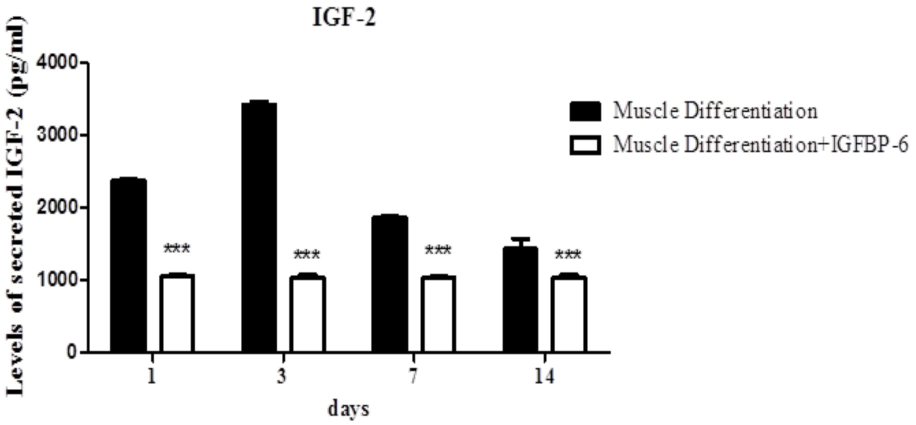

(B)

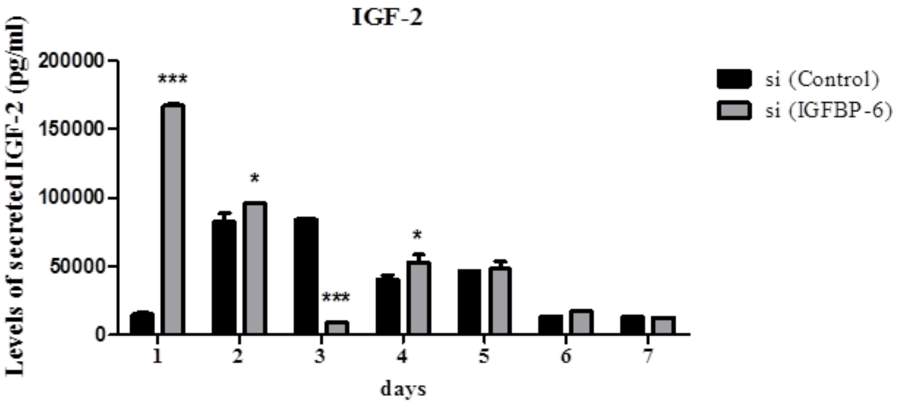

Supplement: Supplementary file 1 — Supplementary Figure 1: PMSC Isolation from 15 weeks preterm placenta. (A) The dissected villous tissue was digested enzymatically and cells were separated using a discontinuous Percoll gradient. Five cell fractions were typically obtained corresponding to five different densities and cells were isolated from layers 3, 4, and 5. (B) Phase contrast images of the isolated PMSCs, from all three layers, grown in culture after 4 weeks. (C) PMSCs from passage 4 of all three layers were positive for CD73 and CD105 (>98%), and were negative for CD117 (<1%) (measured by flow cytometry). Flow cytometry histograms are representative of all 3 layers from 3 placental tissue as they showed the same results. Supplementary Figure 2: IGFBP-6 levels in response to IGFBP-6 supplementation in PMSCs under skeletal muscle differentiation conditions. (A) PMSCs cultured under muscle differentiation conditions showed increased IGFBP-6 protein levels, using western blots, in response to different doses of recombinant human IGFBP-6 protein supplementation with 375 ng/mL and 450 ng/mL having the highest band intensity. (B) IGFBP-6 secretion into the media was increased with the supplementation of recombinant human IGFBP-6 protein (375 ng/mL) that reduced by time and was lower compared to control at day 3. Data is presented as the mean ± SEM of 3 independent experiments. Two-way ANOVA with Bonferroni's multiple comparison test was performed to determine ∗∗∗P < 0.001. Supplementary Figure 3: PMSCs cultured under muscle differentiation conditions showed the formation of multi-nucleated fibers and lower cell count compared to control. (A) At 14 days post-differentiation, PMSCs are immunoreactive for MHC (Red-Alexa 568, λ-568 nm) with cell alignment and multi-nucleated fiber formation (5X). Nuclei, were stained with Hoechst dye (blue, λ=340 nm). (B) PMSCs grown in muscle differentiation media showed multi-nucleated skeletal muscle fiber formation (40X). Black arrows indicate the multi-nucleated mus [file 2348485.f1.pdf]
